# A Case of a C-Stem Fracture at the Head-Neck Junction and a Review of the Literature

**DOI:** 10.1155/2012/158604

**Published:** 2012-12-09

**Authors:** David Morley, Ian Starks, Justin Lim

**Affiliations:** City General Hospital, University Hospital of North Staffordshire, Newcastle Road, Stoke-on-Trent ST4 6QG, UK

## Abstract

We report the first case of a fracture of the standard C-stem in combination with a large metal-on-metal articulation. This occurred at the head-neck junction. Analysis of the fractured stem showed evidence of fatigue failure with possible corrosion. The use of large femoral heads with neck adaptors and narrow tapers should be used with caution, especially in heavy, active patients.

## 1. Introduction

Fracture of the femoral component is a well-documented but rare mode of failure of a total hip arthroplasty (THA), with a reported prevalence ranging from 0.23 [1] to 11 percent [2]. However, with modern stem design this figure is likely to be even lower. The most frequently reported site of fracture is the stem of the femoral component [2–6]. Revision surgery forms the mainstay of treatment. However, in selected cases where the patient is relatively asymptomatic and has reasonable function, a conservative approach may be adopted [5].

Femoral component fracture is almost always preceded by loosening of the component, and it has been suggested that prevention of this loosening is key to preventing stem fracture [7, 8]. 

Numerous factors have been identified as increasing the risk of femoral component fracture. Patient-related factors include male gender, increased weight, increased height, high-activity levels, bilateral hip disease, lumbar spine disease, and the presence of bilateral total hip replacements (THRs) [1, 4, 9]. Surgery-related factors include varus orientation of the stem, poor proximal fixation coupled with rigid distal fixation leading to cantilever bending/fatigue, asymmetrical cement mantle, undersized femoral component, and poor proximal bone support (absence of the calcar) [3, 4, 9]. Factors relating to the prosthesis comprise improper material selection, manufacturing or metallurgic defects, and design flaws leading to stress risers [6, 7, 10].

The last 20 years have seen an increase in the design and use of modular hip systems. These allow the use of mixed-alloy components, for example, combining the wear resistance of a cobalt-alloy femoral head with the flexibility of a titanium-alloy femoral stem [11]. Also, providing the ability to vary neck length and head size independently of the stem reduces stock requirements. However, the increased use of modular hip systems has led to concerns regarding the risk of corrosion at the taper [11–14], leading to implant failure [15, 16]. A study showed that 16–35% of retrieved modular total hip implants showed signs of moderate-to-severe corrosion at the taper [17]. 

We report the first case of a fracture of the standard C-stem. This occurred at the head-neck junction. This case raises concerns over the use of large heads in combination with taper-adapted modular stem systems.

## 2. Case

A 49-year-old male underwent right THR in August 2006. An uncemented metal-on-metal articulation was used—Corail size 10 stem (DePuy, Warsaw, IN) with an ASR 60 millimetre (mm) cup and ASR XL 53 mm head (DePuy, Warsaw, IN). Two years later the patient presented to another institution with pain and decreased range of movement, secondary to subsidence of the femoral stem. Investigations (blood markers, MRI, and bone scan) confirmed aseptic loosening of the femoral component. The patient underwent revision of the right femoral component in January 2009—cemented, high offset, size 5 C-stem (DePuy, Warsaw, IN), and ASR XL 53 mm head (DePuy, Warsaw, IN) with a 9/10 taper adaptor. Initial postoperative check radiographs were satisfactory as was clinical and radiological followup in October 2009. 

The patient was admitted to our hospital as an emergency in March 2010. Following a twisting movement, the patient heard a “click” from his right hip. This was associated with sudden onset of hip pain and an inability to weight bear. Clinically, the leg was shortened and externally rotated. There was no neurovascular deficit. The patient measured 1.85 metre tall and weighed 110 kilograms (kg), resulting in a body mass index of 32.

**Figure 1:**
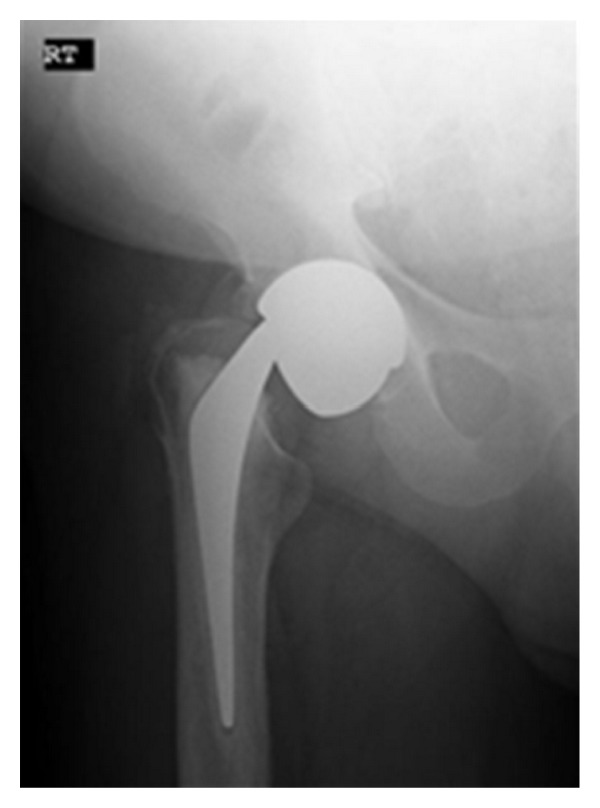
Anteroposterior radiograph demonstrating the fracture of the C-stem at the head-neck junction.

Plain radiographs showed a fracture of the C-stem at the head-neck junction (Figure 1). The inclination of the acetabular component measured 36 degrees.

**Figure 2:**
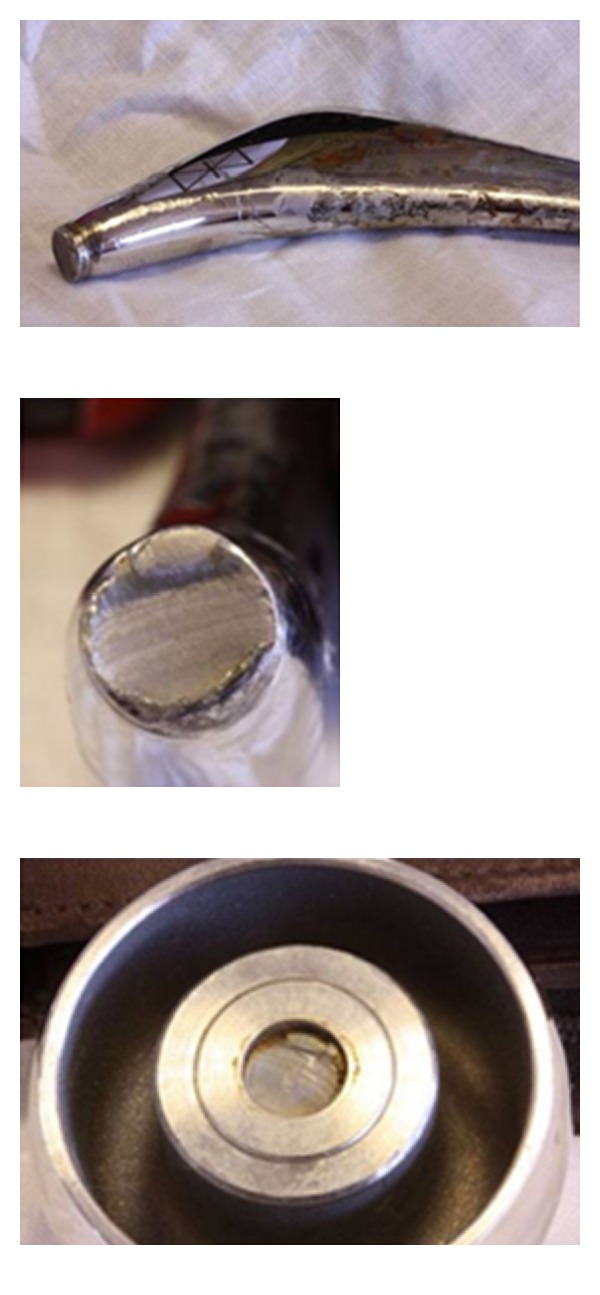
Photographs of the explanted femoral component, demonstrating the fracture at the head-neck junction (left to right: proximal femoral stem, cross section of the fracture site, under surface of the femoral head).

The patient underwent a cement-in-cement revision of the right femoral component through a posterior approach; the rationale is to preserve bone stock in view of the patient's young age. At the time of surgery, there was no evidence to suggest infection. The stem was confirmed to have fractured at the head-neck taper interface (Figure 2). The acetabular component was in excellent condition, with no evidence of scratches or loosening. The orientation was also felt to be satisfactory (inclination approximately 35 degrees, anteversion approximately 10 degrees), and so the cup was not revised. A high offset, size 5 C-stem AMT (DePuy, Warsaw, IN) and ASR XL 53 mm head (DePuy, Warsaw, IN) with a 12/14 taper adaptor was implanted. The 12/14 taper of the C-stem AMT was felt to be more biomechanically advantageous than the 9/10 taper of the C-stem. 

**Figure 3:**
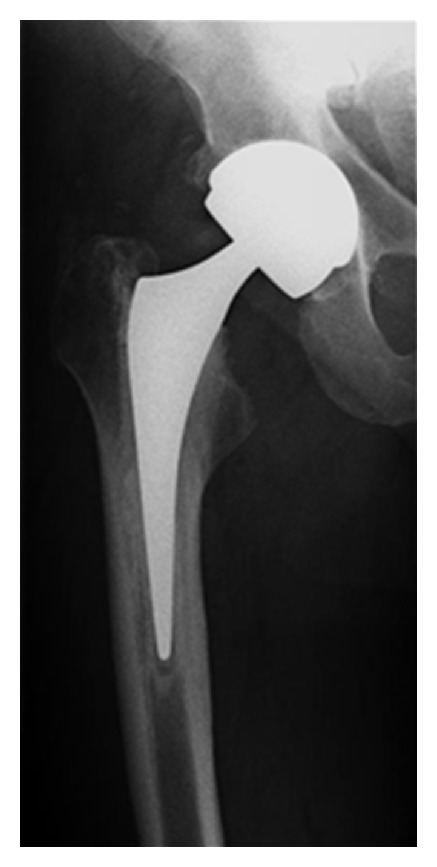
Anteroposterior radiograph following revision surgery using C-stem AMT.

The patient made an uncomplicated recovery and was asymptomatic at 6 weeks followup. Postoperative check radiographs are shown in Figure 3. In January 2011, inflammatory markers and radiographs of the right hip were unremarkable, and chromium and cobalt ion levels showed a downward trend as compared to levels measured in October 2010.

The explanted femoral component was sent to DePuy for further examination. The report showed fatigue failure of the component. The fracture surface was very deformed, particularly around the point of fracture initiation, suggesting that the failure occurred over an extended period of time, allowing separation of the two parts of the taper and damage to occur on the fracture surface/edge. The report also noted signs of metal oxides on the inside of the taper around the fractured edge. This was felt to be compatible with corrosion.

## 3. Discussion

This case is the first report of a fracture of the primary C-stem. Whilst 2 previous C-stem fractures have been reported [18], they occurred in CDH stems. Both patients were female, weighing 83 kg and 89 kg at the time of fracture. The site of fracture in both cases was through the insertion hole, which was postulated to have acted as a stress riser. The authors recommended the use of the primary C-stem instead of the CDH C-stem if possible. The site of fracture in our case is rare, with only a few reported cases in the literature [19]. The case also raises concerns regarding both the use of large heads and head-neck adaptors.

In this case, risk factors for stem fracture include relatively young patient age, male sex, and heavy weight. The fracture of the C-stem occurred at the head-neck junction, where the diameter of the prosthesis is 10 mm. The report on the explanted femoral component in this case suggested fatigue failure, occurring over an extended period of time. The weight of the patient may have led to increased repetitive strain, whilst still being within the normal limit, being placed on the implant. The AMT C-stem used to revise the femoral component has a larger taper than the C-stem (12/14 as opposed to 9/10), thus reducing the risk of fracture.

The use of modular systems brings with it the risk of corrosion at the taper [11, 16, 19]. The crevice formed between the head and neck may act as a corrosion site; the use of a neck adaptor to link a large head to a stem may potentiate the risk of corrosion by introducing an additional taper into the modular system. A case report of a femoral prosthesis fracture in a double-modular system suggested that micromotion at the modular interface between the neck and the stem led to crevice corrosion [20]. One paper reported 2 cases of intergranular corrosion-fatigue failure of modular hip systems, both occurring in heavy, quite active patients at 70 months and 85 months postsurgery [19]. Both implants failed less than one millimetre distal to the taper junction between the head and the stem (outside of the taper). The authors concluded that the reasons for component fracture were intergranular porosity of the implant, an intergranular corrosive attack of the microstructure of the neck, and cyclical loading stresses. 

The increasing use of large femoral heads raises concerns as it is far removed from Charnley's concept of low frictional torque arthroplasty. Torsional forces at the trunnion have been shown to increase as head size increases [21]. The increased frictional torque generated by the use of a large femoral head on a stem with a narrow taper increases the risk of corrosive wear at the head-neck taper [22]. 

In August 2010, DePuy issued a recall of the ASR Articular Surface Replacement and the ASR XL acetabular system. Data from the National Joint Registry showed that the five-year revision rate for the ASR hip resurfacing system is approximately 12% and 13% for the ASR XL acetabular system. Prior to this recall, in a hip requiring revision, if the acetabular component was well seated, revision of the femoral component alone may have been performed, with results comparable to patients undergoing primary THA [23]. One such combination is the ASR XL C-stem, as in our case.

The revision surgery in the above case was performed five months prior to the recall of the ASR Articular Surface Replacement and the ASR XL acetabular system by DePuy. With the current concerns regarding metal on-metal articulations, if this same patient presented to our institution following the recall by DePuy, revision of both the femoral and acetabular components would be performed. We feel that cement-in-cement revision of the femoral component to a C-stem AMT is appropriate. Revision of the acetabular component to an uncemented ceramic-on-ceramic or ceramic-on-polyethylene bearing represent suitable options.

It is not possible to draw firm conclusions from this single case. However, we propose that the cause of the fracture at the head-neck junction in this patient was a combination of cyclical loading stresses, increased frictional torque generated by the use of a large femoral head on a stem with a small diameter taper, and a corrosive element at the taper adaptor.
